# Chemical composition, Fatty acids profile and Biological properties of *Thymus capitatus* (L.) Hoffmanns, essential Oil

**DOI:** 10.1038/s41598-019-56580-y

**Published:** 2019-12-27

**Authors:** Amira Zaïri, Sahar Nouir, Amira Zarrouk, Houda Haddad, Amani Khélifa, Lotfi Achour, Frédéric Tangy, Maher Chaouachi, Mounir Trabelsi

**Affiliations:** 1 Laboratory of Biochemistry, Faculty of Medicine, University of Sousse Tunisia, 4002 Sousse, Tunisia; 2 Laboratory BIOVAL, High Institute of Biotechnology, University of Monastir, 5000 Monastir, Tunisia; 3 Laboratory of cytogenetic, Molecular Biology and Biology of Reproduction; Faculty of Medicine Sousse, University of Sousse Tunisia, 4002 Sousse, Tunisia; 4 Department of Virology, Viral Genomics Unit and Vaccination, Pasteur institute Paris, Paris, France

**Keywords:** Microbiology techniques, Quality of life

## Abstract

*T*. *capitatus* is widely used in traditional medicine in Tunisia. The main goal of this study was to evaluate the phytochemical composition, the fatty acids profile, the antioxidant, antibacterial, and antifungal activities as well as the cytotoxic potential of the essential oil (EO) of this plant. The identification and the quantification of the different constituents of the tested EO was determined by gas chromatography–mass spectrometry (GC-MS). Antioxidant activities were evaluated by spectrophotometric methods and chemical tests. HCT 116 cells were used to evaluate the cytotoxic effect of the EO. The microdilution method was conducted to evaluate the antibacterial activity. Poisoned food method was used to test the antifungal activities against fungi species such *Aspergillus niger* and *Aspergillus flavus*. The EO presented several components, mainly monoterpenes. Results revealed that *T*. *capitatus* EO is not cytotoxic and showed excellent antioxidant activity with a dose dependent manner. Regarding antimicrobial activity, *T*. *capitatus* EO was efficient against all tested bacteria and fungi.

## Introduction

Reactive Oxygen species (ROS) are considered as the most important perpetrators for the spread of multitude of health-related problems. These free radicals may initiate DNA deteriorating and oxidize various molecules such as nucleic acids and proteins^1^. The other concern is infections due to resistant microorganisms. Among the most serious diseases, microbial infections generated by fungal and bacterial strains have long been a topic of interest in the scientific community^2,3^. Thus, there’s an urgent need to develop antibacterial and antifungal agents to treat fungal and bacterial infections. To overcome these opportunistic infections and these sudden outbreaks of oxidative stress, natural products from plant have been intensively explored to provide compounds against a large number of diseases associated to oxidative stress and microorganisms infections^4^. We are interested on the essential oils, which increasingly attract the attention of various fields. Indeed, the EOs are qualified as volatile molecules. They are considered as the products of specialized secretory structures (differentiated parenchyma cells and glandular hairs) and are synthetized via the secondary metabolism of higher plants^5^. These EO are characterized by several biological activities, such as antioxidant, anticonvulsant, analgesic, anxiolytic, and antimicrobial properties^6^.

The Lamiaceae family is widely used as a source of spices and functional ingredients^7^. Within this family, particular consideration was given to the genus Thymus thanks to their food-related biological properties and their aromatic composition. In Tunisia, Thymus species grow at altitudes ranging from 120 to 1100 and are distributed from the sub-humid to the lower arid bioclimates^8^. The pharmacological activity of Thymus EO have been evaluated, including antimicrobial properties and antioxidant activity^9–11^, whereas only minor studies report the bioactivities of the Tunisian *T*. *capitatus* EO against a large panel pathogens including bacteria and fungi. As far as we know no data are available in its cytotoxicity. Accordingly, the purpose of the present work was to evaluate, for the first time, the chemical profile of *T*. *capitatus* EO, (i) its antimicrobial properties (ii) its safety by determining its cytotoxic properties iii) and its fatty acids profile.

## Material and Methods

### Plant material

The Aerial parts of investigated *T*. *capitatus* (leaves) samples were gathered at the complete flowering stage in February and March 2016 from Kairouan, Tunisia (Table 1). The taxonomic identification is authenticated by Ms. Hamdi LAZHAR, the forest engineer of Bouhedma Natural Park, and plant voucher specimens were placed at the 3 herbarium of the Laboratory of Medicinal Plants (INAT). Leaves separated from branches are dehydrated at room temperature for 7 days and slightly blended into fine powders for extractions.

**Table 1:** Collection site of cultivated Thymus capitatus and its eco-geographical characteristics.

Collection site	Geographical Location		
	Longitude (N)	Latitude (E)	Altitude (m)
Kairouan	36°33′84″	10°51″52″	57.6

### The essential oil extraction

Hydro-distillation was used to extract EO from the dried aerial parts of *T*. *capitatus*. Thus, an apparatus of Clevenger type was used. In brief, the extraction was conducted for 3 h by mixing 100 g of plants in 500 mL of distilled water. The extract was dried and concentrated using sodium sulphate and rotatory evaporator under reduced pressure. The EO yield was established by quantity of the obtained oil in mL for 100 g of dried plant. Finally, the pure EO was stored at −4 °C until further analyzed.

### Essential oil analysis

The chemical composition of EO was examined by GC and GC-MS. GC analysis was conducted using a Varian Hp-5890 gas chromatograph equipped with HP5 and Innovax (30 m × 0.25 mm, film thickness 0.25 µm) bonded silica capillary columns and a FID. The temperatures of the injector and detector were 240 °C and 280 °C, respectively. The oven temperature program was 50 °C for 3 min, and then 50–280 °C at 9 °C/min and maintained at 280 °C for 3 min. the carrier gas was nitrogen at a flow rate of 1 ml/min. a volume (0.1 µl) of the diluted sample was injected. The proportion of the constituents was determined by the integration of peak areas.

In addition, a HP 5972/A MS was used to analyze the EO by operating at 70 eV in the same conditions as described above, using the helium at 20 p.s.i. as a carrier gas. The identification of the different compounds was defined by comparison of their retention indexes (determined relatively to the retention times of a series of n-alkanes) with those of standards of the Wiley library search routines^12^, based on fit and purity of mass spectra.

### Cytotoxic activity of extracts

The potential cytotoxicity of *T*. *capitatus* EO was carried out by the MTT based assay and measured in human colon cancer cell line HCT116. In brief, cells were seeded at a density of 5 × 10^3^ per well and were seeded overnight at 37 °C. The culture medium was removed and cells were treated with the EO at concentration ranging from 0 to 2 mg/ml. DMSO was used to dissolve the EO. Further dilution was conducted to reach the desired final concentration. Each concentration was tested in triplicate. After incubation (30 min, 3 h, or 6 h), cells were treated with MTT solution (5 μg/ml in culture medium). To dissolve the dark blue formazan crystals formed by the tetrazolium-formazan reaction, solubilization buffer was added to each well. OD was measured at 595 nm after overnight incubation at 37 °C. The ratio; OD in the test group/OD in the control group) × 100 was calculated to express the percentage of cell survival.

### DPPH Radical scavenging activity

The *T*. *capitatus* EO antioxidant activity was performed by means of the stable DPPH on the basis of a modified method, reported by Kartal *et al*.^13^. Upon its reduction by an antiradical compound, DPPH loose its absorption band at 517 nm. Briefly 180 µl of different concentrations of EO (0.009–0.312 µg/ml) was added to 1620 µl of DPPH, prepared daily. The absorbance was calculated at 517 nm and corresponded to the ability of extract to reduce the DPPH to the yellow-colored diphenylpicrylhydrazine. The ascorbic acid was used as a reference. The antioxidant activity was expressed as IC_50_. A higher antiradical activity of EO corresponds to a lower IC_50_ value. This activity was measured using the following equation: % inhibition = [(A_0_ − A_1_)]/A_0_ × 100.

### Antibacterial activity

Strains used in this work were identified and provided by the department of Clinical Microbiology, Faculty of Medicine of Sousse, Tunisia and were resistant to several conventional antibiotics. They were preserved as follows: *S*. *typhimurium* was grown in LB medium, *E*. *coli*, *S*. *aureus* and *S*. *epidermis* were grown in MH broth. The antibacterial activity of EO sample was performed using the MIC determination method as it was described by Zaïri *et al*.^14^. Briefly, planktonic growths of different strains were tested in the absence and presence of a sample at different concentrations. After an overnight culture, 100 μl of sterile medium were inoculated by each bacterial strain in a 96-well plate to OD_600_ = 0.0001. The samples were used to final concentrations ranging from 0 to 1 mg/ml. Polymyxin (6.25 μg/ml) was used as a positive control. Control wells were prepared were with plant extracts only. Each test was performed in triplicate. Cultures were then incubated with shaking at 37 °C for 24 h. The lowest concentrations that induced 100% inhibition was used to determine MIC values^14^.

### Antifungal activity

The *T*. *capitatus* EO was evaluated fort its antifungal activity against *Aspergillus niger* and *Aspergillus flavus* using a Poisoned food technique as it was described by Kumar *et al*.^15^. Briefly 20 µl of oil sample were added to 10 ml to tepid Potato Dextrose Agar (PDA) medium at appropriate concentrations. Each medium was separately poured into petriplates. At the centre of the Petri dishes were added 5 mm disc of seven day old culture of the *Aspergillus niger* and *Aspergillus flavus*. The plates were incubated at 25 ± 1 °C for seven days. The Petri dishes containing same amount of distilled water and media free from the extract served as control. After incubation, the colony diameter was measured in mm. For each, experiment was performed three times. The percentage of mycelial growth inhibition was calculated using the formula: Antifungal index = (1 − Da/Db) × 100.

### Fatty acids analysis

Total lipids were extracted from the EO according to the method of Moilanen and Nikkari^16^. Boron trifluoride in methanol (14%) was used to transmethylate lipids. Hexane was used to extract the fatty acid methyl esters, which were analyzed by Gas Chromatography (GC) on a Hewlett Packard Model 5890 gas chromatograph (Palo Alto, CA, USA) equipped with a flame ionization detector and a CPSIL-88 column (100 m × 0.25 mm i.d., film thickness 0.20 mm; Varian, Les Ulis, France). The carrier gas was Hydrogen. The oven temperature program was as follows; 60 °C for 5 min, enhanced to 165 °C at 15 °C/min and maintained for 1 min, and then increased to 225 °C at 2 °C/min and then maintained at 225 °C for 17 min. The injector and the detector temperature was detained at 250 °C. Fatty acid were recognized by comparison with synthetic standards. The data were expressed as mg/100 g of oil. This experiment was performed in triplicate.

### Statistical analysis

The statistical analysis was conducted using Statistical Package for the Social Science (SPSS) version 22. The Mann-Whitney U test was used in these analyses. Data were considered statistically different at a P-value of 0.05 or less.

### Ethical approval and informed consent

In this manuscript no experiments were carried out on (i) live vertebrates (or higher invertebrates), (ii) humans or (iii) human samples.

## Results and Discussion

### Essential oil analysis

**Figure 1:**
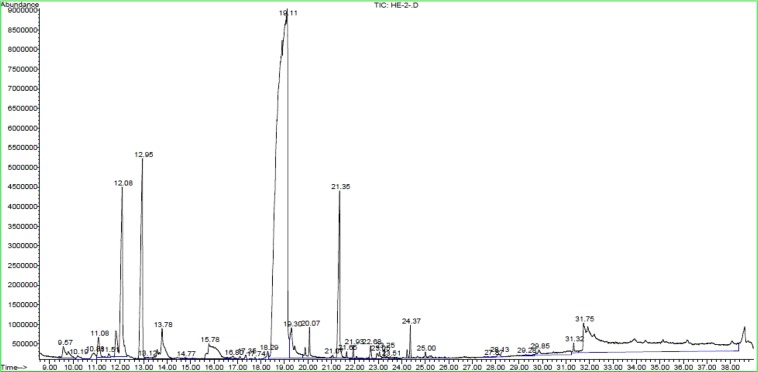
Chromatogram of *T. capitatus* EO.

The essential oil yield of *T*. *capitatus* was 0.82%. Its chemical composition has been identified and quantified based on GC*-*MS. Overall, 25 components were identified, including sesquiterpenes, monoterpenes, and other compounds like, organic acids alcohols and phenols (Table 2, Fig. 1). Hydrocarbons and oxygenated monoterpenes were the predominant classes, while sesquiterpenes and other classes were the least characterized. We noted that the oxygenated monoterpene carvacrol (65.38%) greatly characterizes the *T*. *capitatus* EO being its main constituent. Our results are in concordance with previous study on the EO of *T*. *capitatus* grown in Tunisia, where carvacrol (68.5%), were identified as the major constituent. They also showed that this oil was characterized by the presence of p-cymene, γ-terpinene and β-caryophyllene^17,18^. The high carvacrol content is associated with the dry dwarf-shrub formations of the lowland^19^, and that the content of sesquiterpenes such as b-caryophyllene decreases with increasing altitude^19^. However, reports on different regions of North Africa have shown that major compound Thymus oil species were thymol in Algerian^20^ and Moroccan^21^ samples. Worldwidely, our results are partly in agreement with a recent published report by Gonçalves and co-workers on *T*. *capitatus* EO grown in Sicily who revealed the predominance of oxygenated monoterpens carvacrol (80%) as a major component^22^. Again, according to the literature, the composition of these EOs might be influenced by various factors. The most important are the climate, the soil, and the method of preservation and extraction. Genetic factors, vegetative cycle, altitude as well as the phonological stage can also influence this variability and this certainly contributed to produce a spectacular chemical composition of the oils. It is worth noting that the composition of these volatile oils varies according to the countries, or the places in the same country^23^.

**Table 2:** Chemical composition of *T*. *capitatus* EO.

No	RI	Compound	%
1	935	α-Thujene	0.54
2	940	α-Pinene	0.38
3	991	Myrcene	0.87
4	1019	α.-terpinene	1.11
5	1025	**p-Cymene**	**6.25**
6	1063	**γ-Terpinene**	**6.75**
7	1089	α-terpinolene	0.26
8	1101	Linalool	1.51
9	1179	Terpinen-4-ol	1.40
10	1185	4-Carvomenthenol	0.94
11	1260	Geraniol	0.25
**12**	**1309**	**Carvacrol**	**65.38**
13	1310	Thymol	1.35
14	1358	Eugenol	0.21
15	1408	Carvacryl Acetate	0.45
16	1427	**β-Caryophyllene**	**4.94**
17	1461	α-Humulene	0.10
18	1487	allo*-*aromadendrene	0.18
19	1685	α-Bisabolol	0.35
20	1774	α-Bisabolol oxide A	0.11
21	1815	Hexadecanal	0.14
22	1870	1-Hexadecanol	0.46
23	1879	1-Hexadecanol	0.13
24	1894	Rimuene	0.28
25	1957	Hexadecanoic acid	0.68
		Total identified	95.02
		Unknown	4.98

### *In vitro* toxicity of extracts against human cells

As far as we know, few reports are available on cytotoxicity of a plant extracts before proceeding to their biological activities. Our results revealed that, even when used at high concentrations, *T*. *capitatus* EO is not toxic (Table 3). According to the literature, plants extracts with LC_50_ ≤ 20 g/ml are considered toxic, we can therefore conclude that our extract remains nontoxic^24^. Plants extracts that are toxic to the cells rather than to pathogens, may present no therapeutic value or use. *In vivo* toxicity assays should be tested before a definitive conclusion can be approved. Some studies have reported that the high cytotoxic activity of EO could be mainly attributed to, α-pinene, borneol and β-caryophyllene^25^. Consequently, *T*. *capitatus* EO is less cytotoxic since these compounds were not characterized as its dominant constituents.

**Table 3:** Antioxidant assays (IC_50_ values) *and* Cytotoxicity of *T*. *capitatus* essential oil on HCT-116 (CC_50_ values).

	Cytotoxicity CC_50_ (µg/ml)	DPPH IC_50_
Essential Oil	1482.95 ± 170.98	0.080 ± 0
*T*. *capitatus*		

Data are means ± SD of three independent experiments.

### Antioxidant activity

*T*. *capitatus* EO showed a strong radical scavenging effect, with an IC_50_ = 0.08 mg/mL (Table 3). Our results suggest also that this activity is significantly higher than those of the same local plants. For example, *T*. *capitatus c*ollected from Ras jdir (Tunisia) displayed antioxidant activity at IC_50_ = 340 μg/ml^25^, whereas less than 100 μg/ml of our extracts was enough to reach the same effect. Furthermore, previous studies showed that several other *T*. *capitatus* EO isolated from different countries have been investigated for their antioxidant activity. For example, Tabti and his workers revealed that algerian *T*. *capitatus* EO had a noticeable antioxidant effect with an IC_50_ value of 102 μg/mL^26^. However, Dzami and co-workers showed *T*. *capitatus* EO cultivated in had a potent antioxidant activity with IC_50_ = 119 μg ml^−1^ ^10^. However, none of these studies have showed the safety or the antimicrobial effect of the EO.

Several studies showed that the antioxidant activity could be due to the presence of compounds such as monoterpenes, and oxygenated monoterpenes among them alcohols and phenols. They revealed that phenols and especially carvacrol were confirmed to posses the highest antioxidant activity^27,28^. Our data in antioxidant activity is in the same line with these findings since the percentage of carvacrol, which is a monoterpene phenol, were significantly high (65.38%) in our *T*. *capitatus* EO. Thus, the observed antioxidant activity might be attributed to this compound and to several other components of essential oils, including α-terpinene and γ-Terpinene^25^. However, non-phenolic terpenoids were also reported to have substantial antioxidant potential. In fact, Ruberto *et al*. reported that very high antioxidant activity was observed with monoterpens such as terpinolene, and to a less extent, sabinene which have camparable activity to that of α -tocopherol^29^.

### Antibacterial activity

To date, only few reports have been performed to determine the antibacterial activity of *T*. *capitatus* EO against the strains used in this study. Our results indicate that *T*. *capitatus* EO was efficient when used against all the test organisms (Table 4). Similarly, Ksouri and co-workers showed that antibacterial capacities of *T*. *capitatus* was important against E. coli and S. aureus and exceeded those of T. algeriensis^25^. Other studies revealed that *T*. *capitatus* EO from other countries was more active against bacterial strains. In fact, Dzamic *et al*. reported that the high amount of bioactive compounds in the Lybian *T*. *capitatus* EO, could explain its antibacterial activities. Accordingly, some researchers have described that there is a relationship between the antimicrobial activity and the chemical composition of the EO^23^. For example, the main constituents of the Thymus EOs analyzed in this work were carvacrol, linalool, and 1,8-cineol. These components could be responsible for its strong antibacterial activity in previous studies^30^. Our results showed also that no significant difference in susceptibility between Gram-negative bacteria and Gram-positive bacteria was found, although it was reported that the latter are less sensitive to essential oils^10^. This can be probably due to the fact that the hydrophobic components of EOs may inhibit the functional properties of the bacteria through the disruption of their cell membranes by separating the lipids leading^25^.

**Table 4:** Antibacterial and antifungal activities of *T*. *capitatus* EO.

Essential Oil	Concentration (MIC) in mg/ml of EO				Percentage of mycelial growth inhibition	
	*EC*	*ST*	*SE*	*SA*	*A*. *niger*	*A*. *flavus*
*T*. *capitatus*	0.25^a^	0.12^b^	0.25^a^	0.12^b^	32,67 ± 3,06^b^	73.33 ± 5.79^a^

Value given as mg/ml. Data are means ± SD of three independent experiments. The values with different superscript letters show significantly (p < 0.05) different means. *EC*: *Escherichia Coli; ST*: *Staphylococcus typhimurium*; *SE*: *Staphylococcus epidermis; SA*: *Staphylococcus aureus; A*. *flavus*: *Aspergillus flavus; A*. *niger*: *Aspergillus*. *Niger*.

### Antifungal activity

To the best of our knowledge, no data showed before the antifungal *T*. *capitatus* EO against *A*. *niger* and *A*. *flavus*. Interestingly, our extract was active against fungi with 73.33% mycelial growth inhibition value of 10 µl/mL (Table 4). Furthermore, the evaluation of Thymus EO against other fungal strains have been reported. For example, Ben jabeur *et al*. showed in their study that *T*. *capitatus* (chemotype carvacrol) had the highest antifungal activity followed by *T*. *capitatus* (chemotype thymol) indicating that carvacrol coud have a higher antifungal activity than thymol^17^. The antifungal potential of *T*. *capitatus* EO might be explained to the abundance of phenolic compounds and this result is in accordance with previous data^30^. Almost certainly the high amount of carvacrol in *T*. *capitatus* explains the strong antifungal activity, which approves previous reports indicating a high correlation between phenolic compounds in the EOs and the antimicrobial activity^17^. This activity can be also associated to the presence of minor compounds such as p_cymene and γ-terpinène. Previous reports with regard to that the analysis of the antifungal activity of EOs indicated that phenolic components may interfere with α and ß-glucanases and cell wall enzymes like chitin synthase/chitinase and may attribute for the strong antifungal activity of the EOs^10^. Previous data indicated that antifungal effect of Thymus includes telomerase inhibition, since it enhanced the rate of apoptosis and cell senescence of *Saccharomyces cerevisiae* by accelerating telomere shortening^17^.

### Fatty acids analysis

**Figure 2:**
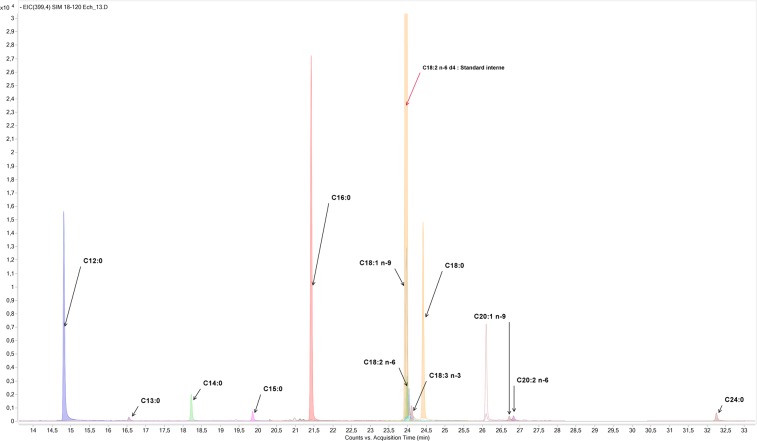
Fatty acids chromatogram of *T. capitatus* EO.

Based on our GC-MS results, where we noticed the presence of a fatty acid (Hexadecanoic acid) and on a recent study conducted by Uchenna *et al*., (2019) and since little is known about fatty acids profile of EOs^31^, it was of interest and for the first time to identify the fatty acids profile of T. capitatus essential oil in order to understand and to explain the antioxidant and the antimicrobial effects of this oil, A total of six monounsaturated fatty acids (MUFA) were identified. The major MUFA was C18:1 n-9 with 0.680 g/kg of the essential oil, followed by C18:1 n-7 and 20:1 n-9. Total polyunsaturated fatty acids (PUFAs) accounted for 0.375 g/kg of the essential oil, with linoleic acid (C18:2 n-6) and α-linolenic (C18:3 n-3) being the two major PUFAs (Table 5, Fig. 2). Since LA and ALA are essential fatty acids, this oil could be considered a novel source of functional foods. These fatty acids are also the precursors of others PUFAs, known to possess a wide range of health benefits especially, eicosapentaenoic acid (C20:5 n-3) and docosahexaenoic acid (DHA; C22:6 n-3)^32^, which could explain even in part the antioxidant effects of this oil.

**Table 5:** Fatty acid composition of *T*. *capitatus EO* (g/Kg of oil).

Fatty acids	g/Kg of essential oil
∑SFA	2.93
12:0	0.703
13:0	0.014
14:0	0.100
15:0	0.063
16:0	1.196
17:0	0.015
18:0	0.822
20:0	0.013
22:0	0.002
24:0	0.001
26:0	0.003
∑MUFA	0.872
16:1 n-7	0.010
16:1 n-9	0.012
18:1 n-9	0.680
18:1 n-7	0.128
20:1 n-9	0.036
22:1 n-9	0.007
∑PUFA	0.375
18:2 n-6	0.231
18:3 n-3	0.094
20:2 n-6	0.037
22:2 n-6	0.013

∑SFA: Total saturated fatty acids; ∑PUFA: Total polyunsaturated fatty acids; ∑MUFA: Total monounsaturated fatty acids.

## Conclusion

On the basis of the results of this work, it’s clearly that T. *capitatus* EO can be used as an easily accessible source of natural antioxidants, antimicrobials compounds and of pharmaceutical application. Devoid of toxicity and rich in phenolic compounds, *T*. *capitatus* could provide considerable benefits for health, not only in the treatment of diseases related to oxidative stress and reactive species production, but also against fungal and bacterial infections. Further analysis could be conducted to determine the whole components of *T*. *capitatus* essential oil and extracts such as the volatile fatty acid profile and/or fatty acids profile of the hexane extract and to evaluate additional biological activities of the essential oil such as the antiviral activity.

## Data Availability

Materials, data and associated protocols are promptly available to readers without undue qualifications in material transfer agreements.
